# Two approaches to account for genotype-by-environment interactions for production traits and age at first calving in South African Holstein cattle

**DOI:** 10.1186/s12711-022-00735-5

**Published:** 2022-06-11

**Authors:** Vincent Ducrocq, Astrid Cadet, Clotilde Patry, Lene van der Westhuizen, Jacob B. van Wyk, Frederick Wilhelm Cornelius Neser

**Affiliations:** 1 Université Paris-Saclay, INRAE, AgroParisTech, GABI, 78350 Jouy-en-Josas, France; 2 Department of Animal Science, University of the Free State, P.O. Box 339, Bloemfontein, 9300 South Africa; 3 ARC-Animal Production Institute, Private Bag X2, Irene, 0062 South Africa

## Abstract

**Background:**

If not accounted for, genotype x environment (G×E) interactions can decrease the accuracy of genetic evaluations and the efficiency of breeding schemes. These interactions are reflected by genetic correlations between countries lower than 1. In countries that are characterized by a heterogeneity of production systems, they are also likely to exist within country, especially when production systems are diverse, as is the case in South Africa. We illustrate several alternative approaches to assess the existence of G×E interactions for production traits and age at first calving in Holsteins in South Africa. Data from 257,836 first lactation cows were used. First, phenotypes that were collected in different regions were considered as separate traits and various multivariate animal models were fitted to calculate the estimates of heritability for each region and the genetic correlations between them. Second, a random regression approach using long-term averages of climatic variables at the herd level in a reaction norm model, was used as an alternative way to account for G×E interactions. Genetic parameter estimates and goodness-of-fit measures were compared.

**Results:**

Genetic correlations between regions as low as 0.80 or even lower were found for production traits, which reflect strong G×E interactions within South Africa that can be linked to the production systems (pasture vs total mixed ration). A random regression model including average rainfall during several decades in the herd surroundings gave the best goodness-of-fit for production traits. This can be related to a preference for total mixed ration on farms with limited rainfall. For age at first calving, the best model was based on a random regression on maximum relative humidity and maximum temperature in summer.

**Conclusions:**

Our results indicate that G×E interactions can be accounted for when genetic evaluations of production traits are performed in South Africa, by either considering production records in different regions as different correlated traits or using a reaction norm model based on herd management characteristics. From a statistical point of view, climatic variables such as average rainfall over a long period can be included in a random regression model as proxies of herd production systems and climate.

**Supplementary Information:**

The online version contains supplementary material available at 10.1186/s12711-022-00735-5.

## Background

South Africa is a large country (1,219,600 km^2^), with a wide variety of climatic conditions that range from a typical Mediterranean climate with winter rainfall in the southwestern corner of the country to a temperate climate in the interior plateau, a subtropical climate in the east and a small area in the northwest with a desert climate, all with summer rainfall. With an average rainfall of only 464 mm, it is a relatively dry country. Most of the country has warm, sunny days and cool nights. Winters in South Africa occur between June and August [1, 2].

The total number of dairy cattle in South Africa is estimated at about 1.37 million [3] with a majority of Holstein cows. These cows are kept under a total mixed ration (TMR) or under a pasture-based production system or a combination of both, depending on the geographical region [4]. Currently between 65 and 75% of the milk production is based on pasture. However, many of these pasture-based systems increasingly incorporate additional feeding such as concentrates or forage crops as hay or silage [5].

In South Africa, breeding values for production traits are computed three times a year [6]. Currently, no consideration is given to the different production environments or climatic conditions when bulls are evaluated. In most national genetic evaluations, local genotype x environment (G×E) interactions are considered as negligible for production traits in dairy cattle [7]. However, if these interactions are present but ignored [8], they can substantially decrease the accuracy of the estimated breeding values (EBV) of bulls, generate biases according to the production environment of their daughters, and ultimately decrease the efficiency of selection at the farmer or country level, i.e. the lower true reliability of the genetic evaluations will reflect negatively on the genetic gain because bull selection for a particular environment is based on inadequate information.

G×E interactions in dairy cattle are most often studied by defining phenotypic expressions in various environments as separate traits [9–15] (say, N traits for N environments) and by estimating the genetic correlations between these N separate traits. An estimate that is significantly different from 1 is interpreted as a signature of a G×E interaction. This is, in particular, the basic assumption underlying the multiple across-country evaluations (MACE) that are routinely calculated by Interbull [10] for national dairy cattle traits. A drawback of the MACE approaches is that the number of (co)variance genetic components (CGC) to be estimated increases quadratically with N. For MACE of milk production in the Holstein breed, this represents 435 CGC for 29 countries [10], which requires significant postprocessing in order to obtain results that are both realistic and consistent.

An intermediate option is to consider only n (< N) underlying traits, in which case the number of GCG increases quadratically only with n. Particular strategies have been proposed to find an optimum number n of underlying traits in international genetic evaluations, such as rank reduction [16, 17] and principal components and factor analyses [18, 19].

At the national level, G×E interactions also exist (e.g., [11–13]), and a particular approach to reveal them consists in adding an interaction term to the traditional quantitative genetic model. For example, a random regression mixed linear model can be built including a reaction norm function of a continuous variable [11, 20, 21], such as a climatic or herd management variable, to reflect the fact that the observed phenotype is the result of a genetic component which differs according to the local environmental conditions [22]. Other strategies consist in constructing structural models in which environmental and genetic variables are used to describe and measure similarities (e.g., between geographical regions). In fact, when modelling G×E interactions, there is a continuum between the two extreme alternatives to describe the link between the genetic component and the environment of the animal, i.e. “one trait stands for one environment” up to “the genetic component is a continuous function of environmental parameters”.

The purpose of this study was to illustrate the wide range of potential genetic evaluation models along this continuum in the particular context of G×E interactions in South African Holstein cows for production traits and age at first calving. The same phenotypes will be described as separate traits in different regions, or by including in the model a reaction norm involving climatic characteristics of the herds averaged over a long period. For simplicity, only first lactation records are considered in this study, to focus more easily on the detection of potential G×E interactions. This is in contrast with the current genetic evaluation model in South Africa, which is based on a 3-lactation fixed regression test-day model [6, 23].

## Methods

Data from 378,782 first lactation Holstein cows recorded over a period of 30 years (1982–2012) were used in the analysis. The traits analyzed were first lactation milk, fat and protein yields (corrected to a 305-day equivalent) and age at first calving (AFC) in months. Only herds in the seven major administrative regions (provinces) with dairy production were considered. Cows were required to have a known sire and an age at first calving between 20 and 40 months. Contemporary groups were defined within herd and included at least 20 cows that were the progeny of sires with at least 20 recorded daughters each in the complete data set. After editing, records from 257,836 first lactation cows from 702 herds were first grouped into four regions according to global climatic characteristics: Western Cape (1), Eastern Cape (2), Free State, Gauteng, North West and Limpopo (3), Kwazulu-Natal (4). Table 1 shows the number of observations and average performances in each region. The data of 10 global climatic variables (yearly rainfall, maximum and minimum temperature, maximum and minimum relative humidity, solar radiation, evapotranspiration, maximum temperature in summer, maximum humidity in summer and maximum solar radiation in summer) from the closest weather station, based on the GPS position of the herd, were averaged over a period of 50 years and added to each herd. To this list, it would have been interesting to add a composite climatic variable such as the average or extreme daily temperature-humidity index (THI) [24] but this was not possible as only yearly averages of temperature and humidity were available. Overall, these variables are used to reflect the long-term climatic characteristics of the herds and not temporary events such as a heat wave or a period of drought. Table 2 presents the average and standard deviation of each climatic variable in the four groups of regions. For all the herds, the feeding system that is categorized in three possibilities (pasture-based, total mixed ration (TMR) or a mixture between the two) had to be known.

**Table 1:** Descriptive statistics of the data set after edits for the four traits studied

Region (code)	N	Mean (± SD)			
		Milk yield (kg)	Fat yield (kg)	Protein yield (kg)	Age at first calving (months)
Western Cape (1)	70,864	8119 ± 2513	297 ± 99	249 ± 80	26.6 ± 3.6
Eastern Cape (2)	40,559	6072 ± 1529	218 ± 59	194 ± 50	28.8 ± 3.7
Free State, Gauteng, North West and Limpopo (3)	75,388	7464 ± 2458	273 ± 93	237 ± 77	28.1 ± 4.0
KwaZulu Natal (4)	71,025	5894 ± 1461	211 ± 53	187 ± 45	28.6 ± 3.8

*N* number of animals; *SD* standard deviation

**Table 2:** Climatic characteristics of the four regions (average over 50 years ± standard deviation)

Climatic variable	Western Cape (1)	Eastern Cape (2)	Free State, Gauteng, North West and Limpopo (3)	KwaZulu Natal (4)
Number of animals	70,864	40,559	75,388	71,025
Rainfall (mm)	554 ± 186	609 ± 107	613 ± 85	853 ± 103
Max temperature (°C)	22.7 ± 0.9	23.0 ± 0.7	23.7 ± 1.2	23.0 ± 1.4
Min temperature (°C)	11.1 ± 0.7	12.2 ± 1.7	8.4 ± 1.3	9.9 ± 1.8
Max relative humidity (%)	88.0 ± 4.4	88.9 ± 2.5	88.9 ± 5.2	91.7 ± 2.9
Min relative humidity (%)	47.2 ± 6.1	49.8 ± 6.3	38.7 ± 5.1	47.3 ± 5.2
Solar radiation (MJ m^−2^ day^−1^)	16.3 ± 0.9	15.6 ± 1.5	19.3 ± 0.7	17.8 ± 1.2
Evapotranspiration (mm)	3.3 ± 0.3	3.1 ± 0.3	3.8 ± 0.3	3.4 ± 0.3
Max temperature in summer (°C)	26.3 ± 1.6	25.4 ± 1.1	27.4 ± 2.2	25.5 ± 1.6
Max humidity in summer (%)	85.2 ± 6.0	91.6 ± 2.9	88.9 ± 7.0	94.5 ± 2.0
Max solar radiation in summer (MJ m^−2^ day^−1^)	25.1 ± 1.6	24.5 ± 1.9	25.0 ± 1.8	25.9 ± 1.6

In a first set of analyses, three linear models (summarized in Table 3) were fitted and analyzed using restricted maximum likelihood, as implemented in the Wombat software [25] The fixed effects included were the same for all analyses: herd-year, calving season (quarter) and age at first calving in months (except for the analyses considering age at first calving as the trait under study).

**Table 3:** Overview of the different models tested

Analysis by region	Univariate	${\mathrm{y}}_{\mathrm{i}}=\sum f+{\mathrm{a}}_{\mathrm{i}}+{\upvarepsilon }_{\mathrm{i}}$
	Multivariate	${\mathrm{y}}_{\mathrm{im}}=\sum f+{\mathrm{a}}_{\mathrm{im}}+{\upvarepsilon }_{\mathrm{im}}$
Analysis including climatic variables	Univariate with heterogeneous residual	${\mathrm{y}}_{\mathrm{im}}=\sum f+{\mathrm{a}}_{\mathrm{i}}+{\upvarepsilon }_{\mathrm{im}}$
	Reaction norm on 1 variable	${\mathrm{y}}_{\mathrm{ir}}=\sum f+{\mathrm{a}}_{\mathrm{i}}+{\uplambda }_{\mathrm{j}}{\mathrm{b}}_{\mathrm{ij}}+{\upvarepsilon }_{\mathrm{im}}$
	Reaction norm on > 1 variables	${\mathrm{y}}_{\mathrm{ir}}=\sum f+{\mathrm{a}}_{\mathrm{i}}+\sum {\uplambda }_{\mathrm{j}}{\mathrm{b}}_{\mathrm{ij}}+{\upvarepsilon }_{\mathrm{im}}$

${\mathrm{y}}_{\mathrm{im}}$ performance of animal $\mathrm{i}$ in region $\mathrm{m}$; $\sum f$ sum of fixed effects (herd-year, calving season and age at first calving for production traits); ${\mathrm{a}}_{\mathrm{im}}$ additive genetic value for animal $\mathrm{i}$ in region $\mathrm{m}$; ${\uplambda }_{\mathrm{j}}$ reaction norm coefficient for climatic variable $\mathrm{j}$; ${\mathrm{b}}_{\mathrm{ij}}$ standardized value of climatic variable $\mathrm{j}$ for animal $\mathrm{i}$; ${\upvarepsilon }_{\mathrm{i}}$ (${\upvarepsilon }_{\mathrm{im}}$): residual for animal $\mathrm{i}$ (in region $\mathrm{m}$)

The first model was a univariate model: the phenotype was considered for the whole population as a unique trait with homogenous variance parameters across environments. This is basically the same assumption as in the current fixed regression test-day model used to estimate breeding values of Holstein cows in South Africa [6, 14].

The second model investigated G×E interactions between regions using a multivariate model: the same phenotypes in each of the four regions were considered as four distinct traits, without preconceived hypotheses about the source of potential G×E interactions. Genetic correlations between traits (here, between regions) were estimated as well as the specific genetic and residual variances from which a heritability could be derived for each region.

The third model was also a multivariate model, but with a reduction of the rank of the correlation matrix that was imposed a priori. The Wombat software [25] reparametrizes the genetic (co)variance matrix in order to force a matrix of rank inferior to the number of traits (i.e., 3, 2 or 1, in our case). This results in a more parsimonious description of the correlation between traits and forces an increased resemblance between regions. The extreme situation is a final rank equal to 1, which implies that all the genetic correlations between regions are equal to 1 but the genetic and residual variances—and therefore heritability—may vary. Goodness-of-fit of the various models were compared using the Bayesian information criterion (BIC), which is recommended when comparing non-nested mixed models with a large number of observations [26]. A lower BIC represents a better model after accounting for the number of parameters to be estimated.

A second group of analyses was set up in order to assess whether the observed differences between regions in terms of production could be related to the climatic conditions that prevailed in each herd. Only milk yield was analyzed and a random regression analysis on climatic variables was used. First, as a base model, a unique (homogeneous) genetic variance and heterogeneous residual variances (one per region) were estimated. This is equivalent to the univariate analysis with a specific residual variance for each region. Then, the model was extended to a random regression model by including each climatic variable as a reaction norm variable: as a result, the genetic parameters of the random regression model included two genetic effects: a fixed term and a regression coefficient on the climatic variable (one at a time). The comparison of these models should reveal which climatic variables contribute to a G×E interaction. Then, two climatic variables were included simultaneously together with the fixed term and the most relevant combination of the two climatic variables was chosen. These random regression analyses were initially run assuming a full rank genetic variance–covariance matrix. This rank was then reduced to 1. In such a case, the first eigenvector of the resulting genetic covariance matrix can be interpreted as the optimal linear combination of the climatic variables describing the environmental component of the genetic variability.

## Results

A summary of the statistics describing first lactation milk, fat and protein yield and age at first calving in the four regions is in Table 1. The mean value of the three production traits differed between the four regions, but for age at first calving, it only differed between Western Cape and the three other regions. The climatic characteristics of the four (groups of) regions are in Table 2. Average rainfall is the variable that varies the most between regions, with the lowest average in the Western Cape.

Genetic parameter estimates for production traits and age at first calving measured in each of the four regions are in Table 4, under six models: two univariate genetic models assuming either a common (U1) or four different (U4) residual variances for the four different regions, and four multivariate models assuming different genetic and residual variances in each region and different assumptions about the rank of the genetic variance matrix (from full (MTR4) to rank 1 (MTR1)). The heritability estimates are generally in agreement with the range of values found in the literature [27–30]. They are lower for protein yield and even more so for fat yield than for milk yield (see Additional file 1: Table S1), possibly because of less accurate on-farm measurements of fat and protein contents compared to milk yield (records are collected by the farmers themselves). Regardless of the trait, rank reductions of the genetic variance matrix did not modify the estimated heritabilities significantly. Heritability estimates varied between regions or models used, especially for production traits. All the genetic correlations estimated using the multivariate models without rank reduction (MTR4) ranged from 0.619 to 0.868 and were usually lower than 0.8. They were all statistically different from 1. These results underline the existence of important G×E interactions, not only as scale effects but also inducing strong bull re-rankings. The effect of rank reduction on genetic correlations was susbtantial, especially for reduction to a rank of 2 (MTR2), or—obviously—1 (MTR1). The inclusion of these interactions in the genetic evaluation model could be more precise by using climatic variables: they appear to define more accurately the variety of environments than the geographical limits, e.g., regions. It appears that including region as a factor of heterogeneity for residual variances already greatly improved the goodness-of-fit of the model.

**Table 4:** Estimates of heritability (in bold) and genetic correlations (below the diagonal) for each region sorted according to Table 1 depending on the trait studied and the model used (± *SE*)

Trait	Univariate analyses		Multivariate analyses			
	Residual variance(s)		Rank 4 (MTR4)	Rank 3 (MTR3)	Rank 2 (MTR2)	Rank 1 (MTR1)
	Unique (U1)	By region (U4)				
Milk yield		**0.194**	**0.234**	**0.233**	**0.238**	**0.231**
		(*0.004*)	(*0.001*)	(*0.009*)	(*0.008*)	(*0.007*)
	**0.254**	**0.326**	**0.748**; **0.364**	**0.778**; **0.365**	**0.765**; **0.365**	**1.000**; **0.363**
	(*0.006*)	(*0.007*)	(*NA*); (*0.002*)	(*0.032*); (*0.013*)	(*0.031*); (*0.012*)	(*NA*); (*0.010*)
		**0.215**	**0.868**; **0.833**; **0.251**	**0.853**; **0.850**; **0.251**	**0.910**; **0.963**; **0.246**	**1.000**; **1.000**; **0.252**
		(*0.005*)	(*0.022*); (*0.026*); (*0.001*)	(*0.024*); (*0.026*); (*0.009*)	(*0.015*); (*0.009*); (*0.008*)	(*NA*); (*NA*); (*0.008*)
		**0.343**	**0.711**; **0.636**; **0.816**; **0.118**	**0.949**; **0.750**; **0.950**; **0.265**	**0.996**; **0.823**; **0.945**; **0.263**	**1.000**; **1.000**; **1.000**; **0.262**
		(*0.007*)	(0.048); (0.060); (0.037); (0.009)	(*0.013*); (*0.034*); (*0.011*); (*0.009*)	(*0.003*); (0*.026*); (*0.012*); (*0.009*)	(*NA*); (*NA*); (*NA*); (*0.008*)
Age at first calving		**0.207**	**0.222**	**0.223**	**0.224**	**0.230**
		*(0.007)*	(0.010)	(*0.010*)	(*0.010*)	(*0.009*)
	**0.177**	**0.170**	**0.619**; **0.184**	**0.644**; **0.175**	**0.594**; **0.175**	**1.000**; **0.160**
	(*0.006*)	(*0.006*)	(*0.056*); (*0.013*)	(*0.052*); (*0.012*)	(*0.052*); (*0.011*)	*(NA*); (*0.009*)
		**0.157**	**0.738**; **0.815**; **0.183**	**0.742**; **0.886**; **0.186**	**0.770**; **0.971**; **0.188**	**1.000**; **1.000**; **0.181**
		(*0.006*)	(*0.038*); (*0.039*); (*0.010*)	(*0.037*); (*0.029*); (*0.010*)	(*0.032*); (0*.010*); (*0.009*)	(*NA*; (*NA*); (*0.008*)
		**0.167**	**0.711**; **0.636**; **0.816**; **0.118**	**0.791**; **0.976**; **0.889**; **0.113**	**0.854**; **0.926**; **0.999**; **0.107**	**1.000**; **1.000**; **1.000**; **0.112**
		(*0.006*)	(*0.048*); (*0.060*); (*0.037*) (*0.009*)	(*0.036*); (*0.010*); (*0.026*; *0.008*)	(*0.025*); (*0.019*); (*0.005*); (*0.006*)	(*NA*); (*NA*); (*NA*); (*0.006*)

*U1* univariate model, one residual variance; *U4* univariate model, four residual variances; *MTRi* multiple traits genetic variance matrix of rank i

*NA* not available due to singularities

Table 5 shows the respective importance of the two eigenvectors of the genetic variance matrix when a reaction norm model was used with one climatic variable included at a time for each studied trait (C1–C10). Given the eigenvector coordinates, it follows that the first eigenvalue roughly represents the part of the total genetic variance that is explained by the average genetic effect regardless of the environment. The second eigenvalue represents the part explained by the reaction norm on the climatic variable.

**Table 5:** Contribution of the second eigenvalue of the genetic covariance matrix, correlation between the two genetic effects (± SE) and goodness-of-fit (BIC was set to 0 for the model without climatic variable) for reaction norm models including a constant and a regression on one climatic variable at a time, in the case of milk yield and age at first calving traits

Climatic variable	Milk yield			Age at first calving		
	Eigenvalues (%)	Correlation (SE)	BIC	Eigenvalues (%)	Correlation (SE)	BIC
C1: Average rainfall	9.93	− 0.637 ± 0.016	− 28.1	8.97	0.236 ± 0.035	202.6
C2: Max temperature	2.64	0.599 ± 0.041	726.9	7.30	− 0.366 ± 0.040	241.5
C3: Min temperature	0.37	− 0.499 ± 0.107	1199.6	3.07	− 0.692 ± 0.037	66.9
C4: Max relative humidity	1.78	− 0.837 ± 0.029	188.3	18.82	0.439 ± 0.023	52.0
C5: Min relative humidity	1.01	− 0.880 ± 0.025	204.0	23.25	− 0.061 ± 0.027	106.2
C6: Solar radiation (SR)	1.13	0.797 ± 0.032	748.5	10.26	0.115 ± 0.039	234.5
C7: Evapotranspiration	1.07	0.867 ± 0.026	315.7	12.14	− 0.119 ± 0.038	206.3
C8: Max temperature in summer	0.99	0.895 ± 0.029	400.9	26.11	0.895 ± 0.029	− 26.3
C9: Max relative humidity in summer	1.06	− 0.911 ± 0.022	65.5	16.09	− 0.346 ± 0.025	− 100.7
C10: Max solar radiation in summer	1.35	0.692 ± 0.048	876.8	16.43	0.692 ± 0.048	110.5

*RH* relative humidity; *SR* solar radiation; *T* temperature

The most important climatic variable for all production traits is the average rainfall, contributing to about 10% of the total genetic variance (C1). In fact, the other climatic variables did not lead to any improvement of the BIC. In contrast, age at first calving was more influenced by the average maximum temperature in summer (C8) and by the average minimum relative humidity (C5), representing about a quarter of the total genetic variance, i.e., the two components related to THI.

To highlight the impact of average rainfall, cows were partitioned into four groups depending on the average rainfall of their herd and a multivariate analysis was performed for milk production (RF4). The results are in Table 6. This analysis showed that a higher level of rainfall was often associated with a lower milk production but also a reduced residual variance and a higher proportion of pasture-based herds (among the herds for which this information was available). However, and quite surprisingly, the heritability was higher in herds with an above-average rainfall level, in spite of their lower average production.

**Table 6:** Herd characteristics, estimates of heritability (in bold) and genetic correlations (below the diagonal of bold figures) for milk yield when herds are grouped according to average rainfall (Model RF4)

Level of average rainfall (mm)	Proportion of herds with pasture^a^	Milk production		
		Mean	Residual standard deviation	Heritability and genetic correlations
> 755	80.5	5962	783	**0.268**
> 659 and < 755	48.8	6070	797	0.909; **0.306**
> 547 and < 659	11.7	7746	1016	0.779; 0.991; **0.257**
< 547	1.8	7989	1062	0.829; 0.858; 0.862; **0.250**

^a^Information on feeding system was available only on a limited fraction of the herds

All the models used were compared according to their goodness-of-fit [BIC, see Table 7 and (Additional file 1: Table S2)]. It can be first concluded that including a heterogeneous residual variance according to the region (U4) strongly increased the goodness-of-fit for the univariate model. However, for milk yield, a reaction norm model including average rainfall (C1) led to a better fit (lower BIC) than models based on regions. Including more climatic variables in the model (C2V, C5V) did not necessarily increase the goodness-of-fit, but greatly increase computation time. The best model was clearly the multivariate model that groups herds according to rainfall level (RF4), with distinct residual variances by region. For age at first calving, the best model was the model that included a reaction norm on maximum relative humidity in summer, followed by the model with a reaction norm on maximum temperature in summer, suggesting again that a model including a reaction norm on temperature-humidity index (THI)—not considered here because only yearly averages of temperature and of humidity were available—could be even better.

**Table 7:** Goodness-of-fit of analyses based on different models for milk production

Model	BIC−BICmin
U1: Univariate	11,190
U4: Univariate + specific residual variances by region	2016
MTR4: Multivariate by region, rank 4	773
MTR1: Multivariate by region, rank 1	967
C1: Reaction norm on average rainfall	745
C2V: Reaction norm on average rainfall and maximum temperature with genetic correlation = 0 and reduced rank	956
C5V: Reaction norm on 5 variables with genetic correlation between “average production” and a linear regression term on climatic variables	967
RF4: Multivariate, grouping herds according to level of rainfall	0^a^

^a^Best model

## Discussion

After more than 50 years of uninterrupted development, genetic (and genomic) evaluations continue to play a fundamental role in the sustainable improvement of dairy cattle. To avoid computational complications, the first animal models were simple, based on strong assumptions, such as cow selection performed only through their sire-to-cow path or successive lactations considered as repetitions of a same trait. These unrealistic assumptions were progressively dropped in many countries with the development of, e.g., multiple-trait animal models and/or models based on test-day records (e.g. [31]). However, some of these initial simplifications are still in use, for example in international evaluations, where daughter yield deviations (DYD) on a standard lactation scale are first derived in order to evaluate bulls on different national scales; these approximations are considered as minor and indispensable in order to properly account for large within-breed G×E interactions between countries, i.e. in international genetic evaluations, a trade-off is necessary between the accurate modeling of national performances and the exploration of G×E interactions.

Our study is another illustration that strong G×E interactions do exist also within country—in our case, South-Africa—with genetic correlations between South-African environments as low as 0.8. In spite of the use of a quite simplistic lactation model, we could demonstrate that genetic correlations for lactation milk yield between regions with different climates could be of the same order of magnitude as the correlation considered in Interbull evaluations between South Africa as a whole and European or North American countries [6]. A likely explanation is the large contrast in average rainfall between farms in different parts of the country, which conditions the management of the cows, i.e. a higher average rainfall favors pasture-based feeding systems. The effect of the feeding systems on genetic parameters has already been demonstrated in previous studies [12, 13, 32]. The use of climatic variables averaged over many years in the genetic part of the model is an indirect way of accounting for the most likely feeding system when this information is not available for all farms. This is very different from, e.g., the inclusion of THI in the model to reflect the short-term influence of extreme climatic conditions (e.g., [33, 34]).

The model that compared herds according to average rainfall over 50 years was statistically better than the model that opposed regions. Interestingly, in contrast with what is classically found in North America or Europe [22] where genetic variance and heritability tend to increase with level of production, the highest heritability was found in the group of herds with substantially lower average production levels (Table 6), due to a much lower estimate of the residual variance. A potential explanation is that large herds based on TMR are grouped and fed according to level of production, with less opportunity to express genetic differences between cows competing for grazing. For clarity, we used a rather trivial lactation model in this study but our conclusions can be extended to more complex genetic or genomic evaluation models, for example based on multivariate random regression models [34].

## Conclusions

This study shows that important G×E interactions exist in Holstein cattle in South Africa that could result in large re-rankings of bulls. In absence of a generalized recording of the farms’s feeding systems, these interactions could be taken into consideration in national genetic evaluations by defining a heterogeneous residual variance by region and a genetic term involving a reaction norm on long-term averages of climatic variables for each herd. In local breeding programs, calculating a different breeding value depending on the environment (region or feeding system) should be considered.

## Supplementary Information

**Additional file 1: Table S1.** Estimates of heritability (in bold) and genetic correlations (below the diagonal) for each region depending the model used (± SE) for Fat yield and Protein yield**. Table S2.** Contribution of the second eigenvalue of the genetic covariance matrix, correlation between the two genetic effects (± SE) and goodness-of-fit (BIC = 0 for the model without climatic variable) for reaction norm models including a constant and a regression on one climatic variable at a time, in the case of fat yield and protein yield; Lower values of BIC indicate a better fit.
