# Supplementary material for: Two approaches to account for genotype-by-environment interactions for production traits and age at first calving in South African Holstein cattle
Source: Genet Sel Evol. 2022 Jun 11;54:43. doi: 10.1186/s12711-022-00735-5 (PMC9188047; doi:10.1186/s12711-022-00735-5)
Supplement: Supplementary file 1 — Additional file 1: Table S1. Estimates of heritability (in bold) and genetic correlations (below the diagonal) for each region depending the model used (± SE) for Fat yield and Protein yield. Table S2. Contribution of the second eigenvalue of the genetic covariance matrix, correlation between the two genetic effects (± SE) and goodness-of-fit (BIC = 0 for the model without climatic variable) for reaction norm models including a constant and a regression on one climatic variable at a time, in the case of fat yield and protein yield; Lower values of BIC indicate a better fit. [file 12711_2022_735_MOESM1_ESM.docx]

**Additional tables**

**Additional file 1: Table S1:** Estimates of heritability (in bold) and genetic correlations (below the diagonal) for each region depending the model used (±SE) for Fat yield and Protein yield

| **Trait** | **Univariate analyses** | | **Multivariate analyses** | | | |
| --- | --- | --- | --- | --- | --- | --- |
|  | **Residual variance(s)** | | **Rank 4 (MTR4)** | **Rank 3 (MTR3)** | **Rank 2 (MTR2)** | **Rank 1 (MTR1)** |
|  | **Unique (U1)** | **By region (U4)** |  |  |  |  |
| Fat yield |  | **0.134** | **0.150** | **0.150** | **0,151** | **0.157** |
|  |  | (0.005) | (*0.008*) | (*0.008*) | (*0.007*) | (*0.006*) |
|  | **0.172** | **0.248** | **0.667 0.308** | **0.635 0.301** | **0.721 0.299** | **1.000 0275** |
|  | *(0.005)* | (0.007) | (*0.048*) *(0.013*) | (*0.048*) (*0.013*) | (*0.038*) (*0.012*) | (*NA*) (*0.010*) |
|  |  | **0.251** | **0.835 0.809 0.169** | **0.862 0.936 0.169** | **0.879 0.965 0.172** | **1.000 1.000 0.173** |
|  |  | (0.005) | (*0.031)* (*0.035)* (*0.008*) | (*0.024*) (*0.015*) (*0.008*) | (*0.021*) (*0.010*) (*0.007*) | (*NA*) (*NA*) (*0.007*) |
|  |  | **0.260** | **0.762 0.697 0.792 0.209** | **0.763 0.745 0.793 0.210** | **0.996 0.657 0.833 0.201** | **1.000 1.000 1.000 0.204** |
|  |  | (0.007) | (0.041) (0.045) (0.034) (0.010) | (*0.039*) (*0.039*) (*0.033*) (*0.010*) | (*0.003*) (0*.043*) (*0.026*) (*0.009*) | (*NA*) (*NA*) (*NA*) (*0.008*) |
| Protein yield |  | **0.166** | **0.198** | **0.199** | **0.199** | **0.197** |
|  |  | *(0.007)* | (0.009) | (*0.009*) | (*0.008*) | (*0.007*) |
|  | **0.216** | **0.280** | **0.660 0.333** | **0.714 0.332** | **0.658 0.331** | **1.000 0.312** |
|  | (*0.006*) | *(0.009)* | (*0.045*) (*0.013*) | (*0.040*) (*0.013*) | (*0.040*) (*0.013*) | *(NA*) (*0.010*) |
|  |  | **0.185** | **0.859 0.795 0.217** | **0.857 0.882 0.215** | **0.904 0.916 0.218** | **1.000 1.000 0.228** |
|  |  | *(0.007)* | (*0.025*) (*0.033*) (*0.009*) | (*0.024*) (*0.019*) (*0.008*) | (*0.015*) (0*.015*) (*0.008*) | (*NA*) (*NA*) (*0.008*) |
|  |  | **0.300** | **0.738 0.643 0.799 0.229** | **0.796 0.584 0.897 0.22** | **0.999 0.674 0.913 0.220** | **1.000 1.000 1.000 0.220** |
|  |  | *(0.008)* | (*0.040*) (*0.047*) (*0.031*) (*0.010*) | (*0.036*) (*0.010*) (*0.017*) (*0.010*) | (*0.001*) (*0.039*) (*0.016*) (*0.009*) | (*NA*) (*NA*) (*NA*) (*0.006*) |

U1: univariate model, one residual variance; U4: univariate model, four residual variances; MTRi: multiple traits genetic variance matrix of rank i

NA: not available due to singularities

**Additional file 1: Table S2** Contribution of the second eigenvalue of the genetic covariance matrix, correlation between the two genetic effects (**±**SE) and goodness-of-fit (BIC = 0 for the model without climatic variable) for reaction norm models including a constant and a regression on one climatic variable at a time, in the case of fat yield and protein yield; Lower values of BIC indicate a better fit.

| **Climatic variable** | **Fat yield** | | | **Protein yield** | | |
| --- | --- | --- | --- | --- | --- | --- |
|  | **Eigenvalues (%)** | **Correlation ±SE** | **BIC** | **Eigenvalues (%)** | **Correlation ±SE** | **BIC** |
| C1: Average rainfall | 9.51 | -0.707±0.017 | -619.8 | 10.49 | -0.672 ±0.016 | -471 |
| C2: Max temperature | 2.84 | 0.663±0.041 | 126.4 | 3.36 | 0.557 ±0.042 | 498 |
| C3: Min temperature | 0.51 | -0.675±0.083 | 515.8 | 0.69 | -0.512 ±0.080 | 875.2 |
| C4: Max RH* | 1.94 | -0.866±0.025 | -351.4 | 1.97 | -0.851 ±0.028 | -144.2 |
| C5: Min RH* | 0.98 | -0.915±0.019 | -485.2 | 0.90 | -0.905 ±0.024 | -93.8 |
| C6: Solar radiation (SR) | 0.87 | 0.877±0.027 | 81.8 | 1.18 | 0.821 ±0.031 | 433 |
| C7: Evapotranspiration | 0.92 | 0.911±0.021 | -313.8 | 0.95 | 0.892 ±0.025 | 14.4 |
| C8: Max T* in summer | 1.33 | 0.898±0.026 | -183.6 | 0.82 | 0.919 ±0.030 | 175.4 |
| C9: Max RH* in summer | 1.21 | -0.920±0.020 | -404 | 1.08 | -0.923 ±0.021 | -264 |
| C10: Max SR in summer | 2.09 | 0.736±0.038 | 154.4 | 1.95 | 0.672 ±0.043 | 568.2 |

RH=relative humidity; SR= solar radiation; T=temperature
